# Measuring the efficacy of routine disinfection methods on frequently used physical therapy equipment

**DOI:** 10.1017/ash.2023.311

**Published:** 2023-09-29

**Authors:** Aaron Barrett, Amanda Graves, Elaine De Jesus, Jennifer Edelschick, Diandrea McCotter, Deverick Anderson, Nicholas Turner, Bobby Warren

## Abstract

**Background:** Frequently used physical therapy (PT) equipment is notably difficult to disinfect due to equipment material and shape, however, the efficacy of standard disinfection of PT equipment is poorly understood. **Methods:** We completed a prospective observational microbiological analysis of fomites used in adult or pediatric PT at Duke University Health System, Durham, North Carolina, from September to December 2022. Predetermined study fomites were obtained after being used during a clinical shift and standard disinfection had been completed by clinical service staff. Fomites were split into 2 halves, left and right, for sampling. Samples were taken with premoistened cellulose sponges processed using the stomacher technique and were incubated on appropriate selective and general medias. We defined antimicrobial-resistant, clinically important pathogens (AMR-CIP) as MRSA, VRE, and MDR-gram-negative isolates, and non–AMR-CIP as MSSA, VSE, and gram-negative species. Study fomites were grouped as follows: (1) pediatric pig toy, (2) walking aids (walkers or canes), (3) balls (medicine, dodge, etc), and (4) other (foam roller, sliding board, etc). **Results:** In total, 47 patients, 61 fomites, and 122 were analyzed. Of the study patients, 24 (51%) were female, 13 (27%) had active infections, and 15 (32%) were on contact precautions. Because fomites were split in half, patients in the left and right study arms were identical. Overall, the median total colony-forming-units (CFU) of study fomites was 1,348 (IQR, 398–2,365): 468 (IQR, 161–1,230) for the left side study arm and 540 (IQR, 102–1,221) for the right study arm (*P* = .45). At the sample level, 52 (43%), 15 (12%), and 37 (30%) of 122 samples harbored any CIPs, AMR CIPs, or non-AMR CIPs, respectively. At the fomite level, 27 (44%), 5 (8%), 15(25%), and 7 (11%) of 61 fomites harbored any CIPs, only AMR-CIPs, only non-AMR CIPs, or both AMR and non-AMR CIPs, respectively. Generally, therapy balls were the most contaminated study fomites (n = 2,237; IQR, 1,425–2,658), and walking aids were most frequently contaminated with any CIPs (n = 26, 72%), AMR CIPs (n = 8, 22%), and non-AMR CIPs (n = 15, 47%). **Discussion:** Following routine disinfection, frequently used PT equipment remained heavily contaminated and harbored AMR and non-AMR CIPs, supporting the notion that PT equipment is difficult to disinfect via standard disinfection. Additionally, left-, and right-side fomite divisions had similar pathogens, suggesting that this sampling model of intrapatient comparisons may be helpful for resolving case-mix issues in future studies. Future work should focus on PT-specific enhanced disinfection strategies to improve the disinfection of PT equipment.

**Figure:**
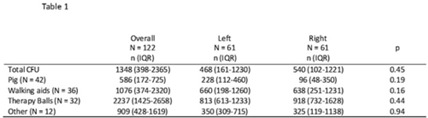

**Figure:**
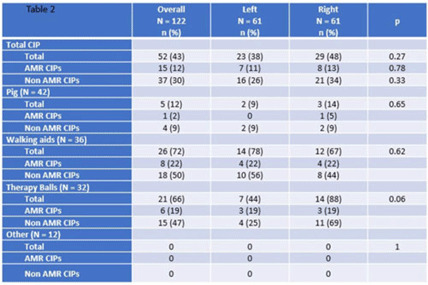

**Financial support:** This study was funded by PURioLABS.

**Disclosures:** None

